# Serum KL-6 levels predict the occurrence and severity of treatment-related interstitial lung disease in lung cancer

**DOI:** 10.1038/s41598-023-45170-8

**Published:** 2023-10-23

**Authors:** Hwa Kyung Park, Chang-Seok Yoon, Young-Ok Na, Jae-Kyeong Lee, Hyung-Joo Oh, Ha-Young Park, Bo-Gun Kho, Tae-Ok Kim, Hong-Joon Shin, Yong-Soo Kwon, In-Jae Oh, Yu-Il Kim, Sung-Chul Lim, Young-Chul Kim, Cheol-Kyu Park

**Affiliations:** 1 Lung Cancer Center, Chonnam National University Hwasun Hospital, Hwasun, Jeollanam-do Republic of Korea; 2 Department of Internal Medicine, Chonnam National University Medical School, Hwasun, Jeollanam-do Republic of Korea; 3 Department of Internal Medicine, Chonnam National University Hospital, Gwangju, Republic of Korea; 4 Department of Internal Medicine, Chonnam National University Bitgoeul Hospital, Gwangju, Republic of Korea

**Keywords:** Respiratory signs and symptoms, Non-small-cell lung cancer, Small-cell lung cancer, Diagnostic markers, Predictive markers, Prognostic markers, Risk factors

## Abstract

In this study, we aimed to investigate the feasibility of serum Krebs von den Lungen-6 (KL-6) as a potential biomarker for treatment-related ILD (TR-ILD) in lung cancer. We recruited patients with lung cancer in whom KL-6 was measured to differentiate between pneumonia and ILD (category 1), diagnose and assess the severity of suspicious ILD (category 2), or evaluate baseline levels before cancer treatment (category 3). Among 1,297 patients who underwent KL-6 testing, 422 had lung cancer, and TR-ILD was detected in 195 patients. In categories 1–2, median KL-6 level was higher in drug-induced ILD or acute exacerbation of underlying ILD than in no ILD or radiation-induced pneumonitis, and it was correlated with the severity of TR-ILD. High KL-6 level (cut-off: > 436U/mL) was an independent risk factor for severe TR-ILD, and low KL-6 level with high procalcitonin level (> 0.5 ng/mL) could exclude severe TR-ILD. Patients with severe TR-ILD had worse overall survival than those without, whereas high baseline KL-6 level was associated with worse survival, especially in patients without severe TR-ILD. Therefore, serum KL-6 may be a surrogate marker for predicting the occurrence and assessing the severity of TR-ILD at the time of suspected ILD and before lung cancer treatment.

## Introduction

Lung cancer has the highest mortality rates relative to other malignancies worldwide, including in South Korea^1, 2^. However, the development of several robust therapeutic agents for treating cancer, including targeted agents and immune checkpoint inhibitors (ICIs), has led to improvements in survival in patients with advanced-stage lung cancer^3^. These novel therapies have also led to survival benefits in non-advanced lung cancer. Adjuvant treatment with osimertinib, a third-generation epidermal growth factor receptor (EGFR) tyrosine kinase inhibitor, was recently incorporated into the standard treatment of EGFR-mutated early-stage non-small-cell lung cancer (NSCLC)^4^. The successes of randomized clinical trials of anti-programmed death-1 (PD-1) or PD-ligand 1 (PD-L1) monoclonal antibodies have presented a new horizon of NSCLC treatment, with options for neoadjuvant treatment^5^, adjuvant treatment^6, 7^, and consolidation treatment after chemoradiation therapy (CRT)^8^.

However, as survival rates and corresponding durations of treatment increase, lung cancer treatment can lead to additional long-term exposure to adverse events, particularly pneumonitis. In one study, among patients with locally advanced NSCLC who received concurrent CRT (CCRT), 83% and 34% developed pneumonitis and symptomatic pneumonitis, respectively^9^. According to a real-world multi-institutional cohort study, 22% of patients who received PD-1/PD-L1 inhibitors suffered from pneumonitis, and patients with pneumonitis showed an increased risk of death (hazard ratio [HR]: 2.34, 95% confidence interval [CI] 1.47–3.71). Therefore, minimizing the treatment interruption necessitated by treatment-related adverse events is crucial to improve treatment compliance and extend treatment duration and survival. Several biomarkers have been investigated to predict the development of treatment-related pneumonitis induced by radiation^10^ and ICIs^11, 12^ in lung cancer. However, few studies have shown validated blood-based predictors of the extent, severity, and prognosis of pneumonitis^13^, relevant to its biological variations.

Krebs von den Lungen-6 (KL-6) is a mucinous high-molecular-weight glycoprotein expressed on the surface of type II alveolar epithelial cells. KL-6 diffuses into the bloodstream following damage to the epithelial cell membranes of type II alveolar epithelial cells; this results in elevated serum KL-6 levels, which reflect the extent of damage and regeneration of type II pneumocytes^14, 15^. According to a previous meta-analysis and retrospective study, higher KL-6 levels correlate with more severe or acutely exacerbated interstitial lung disease (ILD), making KL-6 a biomarker of poor prognosis^16, 17^. In several studies of patients with lung cancer, KL-6 has been used to predict the development of post-treatment pneumonitis, such as drug-induced ILD^18^, including ICI-related interstitial pneumonitis^19^, radiation-induced pneumonitis (RP)^20^, and exacerbation of underlying idiopathic ILD with lung cancer^21^, which can be considered treatment-related ILD (TR-ILD). However, no previous study has validated KL-6 in distinguishing TR-ILD from pneumonia or non-ILD events at the onset of adverse events similar to ILD after cancer treatment^19^, which can be encountered in actual clinical practice.

On the basis of the unmet need for developing biomarkers for treatment-related adverse events, we aimed to evaluate the usefulness of KL-6 as a biomarker of TR-ILD during or after lung cancer treatment.

## Results

### Baseline characteristics and serum KL-6 levels

**Figure 1:**
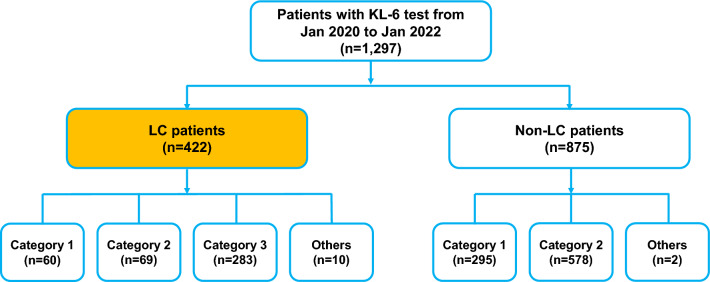
Flow chart of patient enrollment. Serum KL-6 was tested in category 1 for differentiating between pneumonia and ILD, in category 2 for diagnosing and assessing the severity of events suspected to be ILD, and in category 3 for evaluating the baseline level before the initiation of cancer treatment. LC: Lung cancer.

The flow of patient enrollment and a summary of the baseline characteristics of patients with lung cancer are presented in Fig. 1 and Table 1, respectively. We recruited 1,297 patients who were tested for serum KL-6, among whom 422 (32.5%) had lung cancer. Most patients with lung cancer had a smoking history (85.3%), and 50.7% had underlying chronic obstructive pulmonary disease. More than half of the patients (57.8%) were diagnosed with stage IV disease, and most patients had received CCRT (38.4%) or systemic chemotherapy (42.6%) as the initial lung cancer treatment. TR-ILD was detected in 195 patients (46.2%), with RP (61.0%) representing the most common subtype. We divided the study patients into three groups based on the purpose and timing of KL-6 measurement: differentiating between pneumonia and ILD (category 1), diagnosing and assessing the severity of suspicious ILD (category 2), and evaluating the baseline level before initiating cancer treatment such as RT or ICI administration (category 3). For categories 1 and 2, serum KL-6 levels were tested at the onset of the relevant events. The most common indication for serum KL-6 testing was category 3. Pre-existing ILD was identified in 44 patients (10.4%), and patients with category 2 had the highest proportion of underlying ILD (36.2%), mostly idiopathic pulmonary fibrosis.

**Table 1:** Baseline characteristics of patients with lung cancer.

Characteristic	Total (*n* = 422)	Category 1 *(n* = 60)	Category 2 (*n* = 69)	Category 3 (*n* = 283)	Unknown (*n* = 10)
Age, years	70 (36–86)	71 (52–82)	71 (47–85)	69 (36–86)	72 (62–78)
Sex					
Female	48 (11.4)	8 (13.3)	9 (13.0)	29 (10.2)	2 (20.0)
Male	374 (88.6)	52 (86.7)	60 (87.0)	254 (89.8)	8 (80.0)
Smoking					
Never smoker	62 (14.7)	7 (11.7)	10 (14.5)	45 (15.9)	0 (0.0)
Current smoker	153 (36.3)	15 (25.0)	27 (39.1)	105 (37.1)	6 (60.0)
Ex-smoker	207 (49.0)	38 (63.3)	32 (46.4)	133 (47.0)	4 (40.0)
Comorbidity					
ILD	44 (10.4)	5 (8.3)	25 (36.2)	14 (4.9)	0 (0.0)
IPF	34 (77.3)	2 (40.0)	22 (88.0)	10 (71.4)	–
Non-IPF	10 (22.7)	3 (60.0)	3 (12.0)	4 (28.6)	–
COPD	214 (50.7)	24 (40.0)	34 (49.3)	148 (52.3)	8 (80.0)
ECOG PS score					
0	15 (3.5)	1 (1.7)	0 (0.0)	13 (4.6)	1 (10.0)
1	298 (70.6)	34 (56.7)	45 (65.2)	212 (74.9)	7 (70.0)
2	97 (23.0)	17 (28.3)	22 (31.9)	56 (19.8)	2 (20.0)
3	8 (1.9)	5 (8.3)	1 (1.4)	2 (0.7)	0 (0.0)
4	4 (1.0)	3 (5.0)	1 (1.4)	0 (0.0)	0 (0.0)
Pulmonary function					
FEV1, L (*n* = 370)	2.03 (0.52–3.80)	2.04 (0.66–3.67)	2.05 (0.90–3.32)	2.03 (0.52–3.80)	1.87 (1.54–3.72)
FEV1, % (*n* = 370)	71.1 (20.1–124.5)	73.1 (25.6–124.5)	73.7 (30.2–103.3)	70.7 (20.1–120.6)	68.7 (58.0–106.6)
FVC, L (*n* = 370)	2.86 (1.11–6.24)	2.84 (1.41–4.95)	2.73 (1.11–5.81)	2.91 (1.26–6.24)	3.03 (2.06–4.16)
FVC, % (*n* = 370)	71.4 (30.8–116.8)	72.9 (32.9–108.8)	70.2 (33.4–116.8)	71.2 (30.8–110.6)	77.2 (51.9–85.1)
DLCO, % (*n* = 324)	66.6 (24.9–133.0)	66.5 (36.7–133.0)	60.5 (24.9–110.7)	67.8 (29.2–126.2)	66.9 (54.3–98.6)
Serum CEA, ng/mL (*n* = 373)	5.50 (0.88–11,450.37)	4.77 (0.93–78.67)	7.40 (1.13–801.20)	5.36 (0.88–11,450.37)	9.50 (1.75–2248.70)
Serum CRP, mg/dL (*n* = 317)	1.42 (0.02–52.57)	3.78 (0.02–34.26)	0.92 (0.02–52.57)	1.44 (0.03–31.37)	0.36 (0.07–6.64)
Serum PCT, ng/mL (*n* = 73)	0.20 (0.03–2.87)	0.18 (0.04–1.48)	0.23 (0.04–2.33)	0.20 (0.03–2.87)	0.22 (NA)*
Histology					
Adenocarcinoma	154 (36.5)	21 (35.0)	32 (46.4)	99 (35.0)	2 (20.0)
Squamous cell carcinoma	174 (41.2)	25 (41.7)	22 (31.9)	120 (42.4)	7 (70.0)
NSCLC, NOS	17 (4.0)	3 (5.0)	2 (2.9)	12 (4.2)	0 (0.0)
Small cell lung carcinoma	77 (18.2)	11 (18.3)	13 (18.8)	52 (18.4)	1 (10.0)
Stage (TNM 8th)					
Early (I-III, LD)	178 (42.2)	27 (45.0)	37 (53.6)	108 (38.2)	6 (60.0)
Advanced (IV, ED)	244 (57.8)	33 (55.0)	32 (46.4)	175 (61.8)	4 (40.0)
Driver mutations					
EGFR (tested, *n* = 191)	34/191 (17.8)	10/28 (35.7)	9/35 (25.7)	15/126 (11.9)	0/2 (0.0)
ALK (tested, *n* = 186)	14/186 (7.5)	1/28 (3.6)	3/31 (9.7)	10/125 (8.0)	0/2 (0.0)
ROS1 (tested, *n* = 166)	2/166 (1.2)	0/23 (0.0)	1/27 (3.7)	1/114 (0.9)	0/2 (0.0)
PD-L1 IHC, SP263 (tested)	(*n* = 312)	(*n* = 41)	(*n* = 49)	(*n* = 214)	(*n* = 8)
TPS < 1%	120 (38.6)	15 (36.6)	26 (53.1)	74 (34.6)	5 (62.5)
TPS ≥ 1%, < 50%	90 (28.8)	13 (31.7)	9 (18.3)	66 (30.8)	2 (25.0)
TPS ≥ 50%	102 (32.6)	13 (31.7)	14 (28.6)	74 (34.6)	1 (12.5)
PD-L1 IHC, 22C3 (tested)	(*n* = 157)	(*n* = 24)	(*n* = 31)	(*n* = 99)	(*n* = 3)
TPS < 1%	39 (24.8)	6 (25.0)	8 (25.8)	24 (24.2)	1 (33.3)
TPS ≥ 1%, < 50%	56 (35.7)	5 (20.8)	11 (35.5)	39 (39.4)	1 (33.3)
TPS ≥ 50%	62 (39.5)	13 (54.2)	12 (38.7)	36 (36.4)	1 (33.3)
Initial therapy of lung cancer					
Operation	59 (14.0)	11 (18.3)	10 (14.5)	37 (13.1)	1 (10.0)
CCRT	162 (38.4)	23 (38.3)	29 (42.0)	107 (37.8)	3 (30.0)
Systemic chemotherapy	180 (42.6)	22 (36.7)	22 (31.9)	132 (46.6)	4 (40.0)
SBRT or RT alone	12 (2.8)	2 (3.3)	6 (8.7)	4 (1.4)	0 (0.0)
Palliative treatment	3 (0.7)	0 (0.0)	1 (1.4)	2 (0.7)	0 (0.0)
Supportive care	6 (1.4)	2 (3.3)	1 (1.4)	1 (0.4)	2 (20.0)
Immune-related adverse events (investigated, *n* = 270)	53/270 (19.6)	8/19 (42.1)	9/15 (60.0)	36/236 (15.3)	0/0 (0.0)
ILD	24 (45.2)	5 (62.5)	8 (88.9)	11 (30.6)	–
Non-ILD	29 (54.8)	3 (37.5)	1 (11.1)	25 (69.4)	–
ILD type	195 (46.2)	38 (63.3)	61 (88.4)	96 (33.9)	0 (0.0)
DILD	51 (26.2)	19 (50.0)	19 (31.2)	13 (13.6)	–
RP	119 (61.0)	18 (47.4)	26 (42.6)	75 (78.1)	–
AE-ILD	25 (12.8)	1 (2.6)	16 (26.2)	8 (8.3)	–
Survival					
Live	244 (57.8)	23 (38.3)	41 (59.4)	175 (61.8)	5 (50.0)
Death	73 (17.3)	18 (30.0)	13 (18.8)	42 (14.8)	0 (0.0)
Lost follow-up or hopeless discharge	105 (24.9)	19 (31.7)	15 (21.7)	66 (23.3)	5 (50.0)

Values are presented as medians (ranges) or numbers (%).

*ILD* Interstitial lung disease, *IPF* Idiopathic pulmonary fibrosis, *COPD* Chronic obstructive pulmonary disease, *ECOG* Eastern Cooperative Oncology Group, *PS* Performance status, *FEV1* Forced expiratory volume within 1 s, *FVC* Forced vital capacity, *DLCO* Diffusing capacity of lungs for carbon monoxide, *CEA* Carcinoembryonic antigen, *CRP* C-reactive protein, *PCT* Procalcitonin, *NA* Not available, *NSCLC* Non-small cell lung carcinoma, *NOS* Not otherwise specified, *LD* Limited disease (for small cell lung cancer), *ED* Extensive disease (for small cell lung cancer), *EGFR* Epidermal growth factor receptor, *ALK* Anaplastic lymphoma kinase, *ROS1* ROS proto-oncogene 1, *PD-L1* Programmed death-ligand 1, *IHC* Immunohistochemistry, *TPS* Tumor proportion score, *CCRT* Concurrent chemoradiation therapy, *SBRT* Stereotactic body radiation therapy, *RT* Radiation therapy, *DILD* Drug-induced ILD, *RP* Radiation-induced pneumonitis, *AE-ILD* Acute exacerbation of underlying ILD.

*Only case.

The baseline characteristics of patients without lung cancer and comparison with those of patients with lung cancer are described in Supplementary Table S1 online. In the overall population, the median serum KL-6 level (U/mL) was higher among patients who developed ILD (*n* = 865) than among those without ILD (*n* = 432) (600.7 vs. 326.7; *p* < 0.001) and higher among patients without lung cancer (*n* = 875) than among those with lung cancer (*n* = 422) (556.5 vs. 369.3; *p* < 0.001) (see Supplementary Fig. S1a–b online). However, in categories 1 and 2, there were no significant differences in serum KL-6 levels between patients with (*n* = 129) and without lung cancer (*n* = 873) (see Supplementary Fig. S1c online).

**Figure 2:**
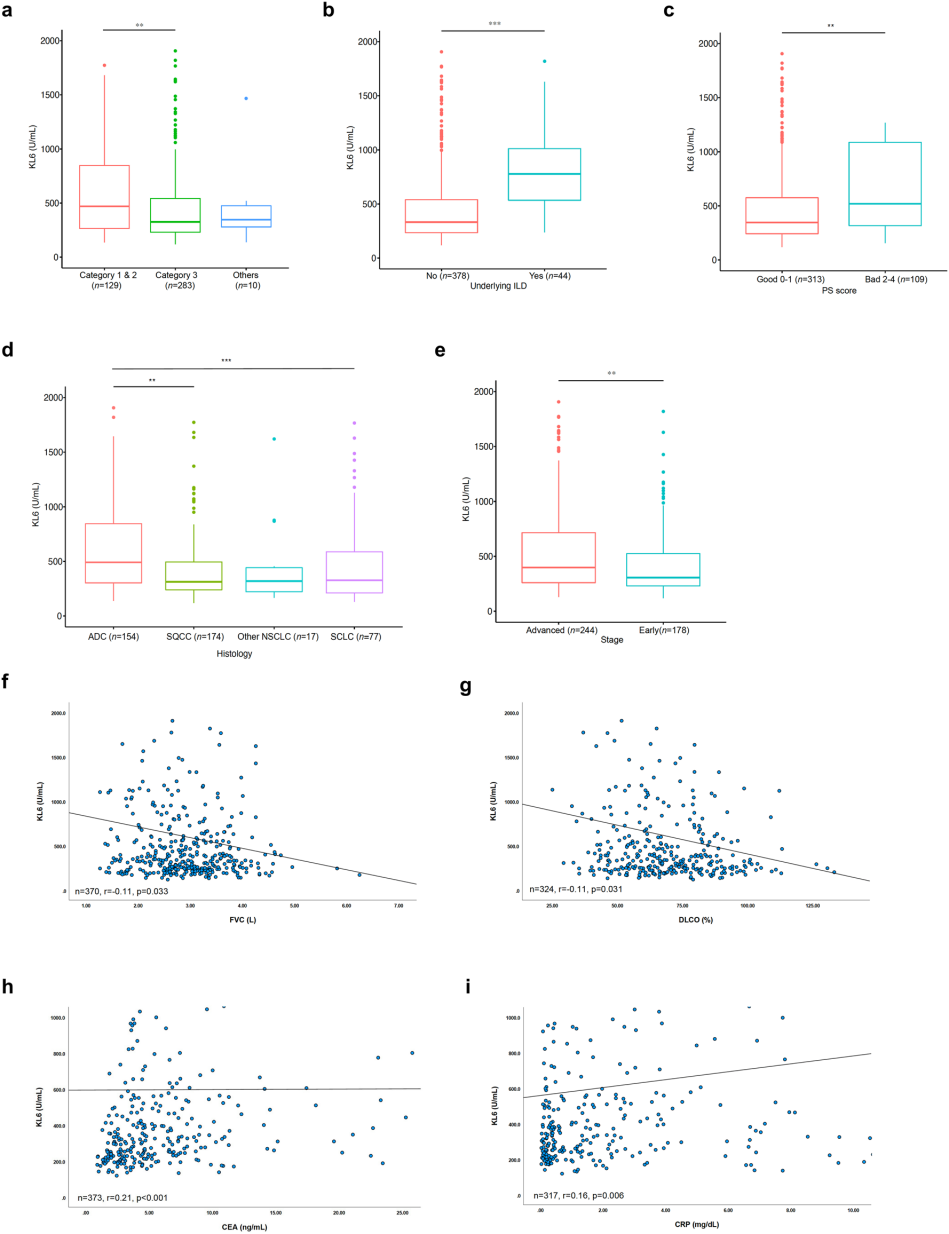
Serum KL-6 in patients with lung cancer. (**a**–**e**) The box-and-whisker plots show differences in median KL-6 levels according to (**a**) categories of serum KL-6 test, (**b**) the presence of underlying ILD, (**c**) ECOG PS score, (**d**) histology, and (e) stage in patients with lung cancer. (**f**–**i**) The scatter plots show the correlation between serum KL-6 level and (**f**) FVC (L) (*r* = -0.11, *p* = 0.033), (**g**) DLCO (%) (*r* =  − 0.13, *p* = 0.016), (**h**) serum CEA (*r* = 0.21, *p* < 0.001) and (**i**) serum CRP level (*r* = 0.16, *p* = 0.006). ^**^p < 0.01; ^***^p < 0.001. *ILD* Interstitial lung disease, *ECOG* Eastern Cooperative Oncology Group, *PS* Performance status, *ADC* Adenocarcinoma, *SQCC* Squamous cell carcinoma, *NSCLC* Non-small cell lung carcinoma, *SCLC* Small cell lung carcinoma, *FVC* Forced vital capacity, *DLCO* Diffusing capacity of lungs for carbon monoxide, *CEA* Carcinoembryonic antigen, *CRP* C-reactive protein.

Among patients with lung cancer, the median serum KL-6 levels were significantly higher in categories 1 and 2 (combined) than in category 3 (492.2 vs. 333.0; *p* = 0.019) (Fig. 2a), higher in patients with underlying ILD than in those without ILD (821.3 vs. 335.8; *p* < 0.001) (Fig. 2b), and lower in patients with better Eastern Cooperative Oncology Group (ECOG) performance status (PS) scores (0 to 1) than in those with worse scores (2 to 4) (330.7 vs. 506.8; *p* < 0.001) (Fig. 2c). Patients with adenocarcinoma had a higher median serum KL-6 level than those with squamous cell carcinoma (519.4 vs. 317.8; *p* < 0.001) and small cell carcinoma (519.4 vs. 322.4 ; *p* < 0.001) (Fig. 2d), and patients with advanced-stage disease had a higher median serum KL-6 level than those with early-stage lung cancer (409.1 vs. 321.0; *p* = 0.007) (Fig. 2e). In addition, serum KL-6 levels had a negative correlation with forced vital capacity (FVC; L) (*r* =  − 0.11; *p* = 0.033) (Fig. 2f) and diffusing capacity of the lungs for carbon monoxide (DLCO; %) (*r* =  − 0.11; *p* = 0.031) (Fig. 2g), as well as a positive correlation with carinoembryonic antigen (CEA; ng/mL) (*r* = 0.21; *p* < 0.001) (Fig. 2h) and C-reactive protein (CRP; mg/dL) (*r* = 0.16, *p* = 0.006) (Fig. 2i).

### Serum KL-6 levels and TR-ILD in lung cancer

**Figure 3:**
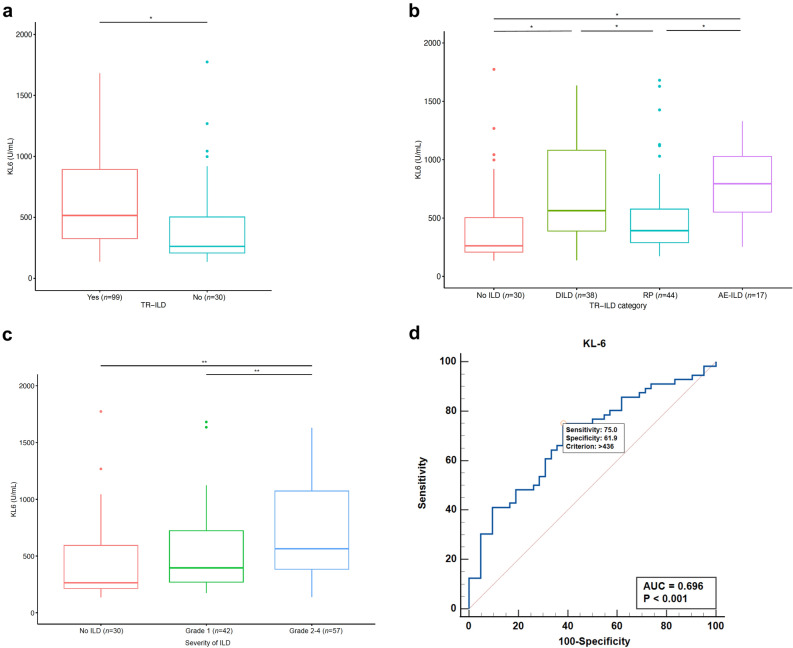
Serum KL-6 and TR-ILD in patients with lung cancer (categories 1 and 2). (**a**–**c**) The box-and-whisker plots show differences in median KL-6 levels according to (**a**) the presence of TR-ILD, (**b**) the subtype, and (**c**) the severity of TR-ILD. (**d**) ROC curve analysis of cut-off serum KL-6 value for predicting severe TR-ILD. ^*^p < 0.05, ^**^p < 0.01. *TR-ILD* Treatment-related interstitial lung disease, *DILD* Drug-induced ILD, *RP* Radiation-induced pneumonitis, *AE-ILD* Acute exacerbation of underlying ILD, *ILD* Interstitial lung disease, *ROC* Receiver operating characteristic, *AUC* Area under the curve.

In our study, there were 373 patients with lung cancer treated with radiation therapy (RT; *n* = 273) or ICIs (*n* = 276), and 195 patients developed TR-ILD (Table 1). In categories 1 and 2, patients with TR-ILD (*n* = 99) had a significantly higher median serum KL-6 level than those without TR-ILD (*n* = 30) (531.0 vs. 261.8; *p* = 0.045) (Fig. 3a). In terms of the TR-ILD subtypes, the median serum KL-6 level was higher among patients with drug-induced ILD (DILD) than among those without ILD (543.0 vs. 261.8; *p* = 0.040) or among those with RP (vs. 391.6; *p* = 0.047). Patients with acute exacerbation of underlying ILD (AE-ILD; 963.6 U/mL) also had a higher median serum KL-6 level than those without ILD (*p* = 0.023) or those with RP (*p* = 0.028), although there was no significant difference compared to patients with DILD (Fig. 3b). Furthermore, the median serum KL-6 level correlated with the severity of TR-ILD (395.8 in grade 1 vs. 633.0 in grades 2–4; *p* = 0.007) (Fig. 3c). The cut-off serum KL-6 value for predicting severe TR-ILD (grade 2 or higher) in categories 1 and 2 was determined by receiver operating characteristic (ROC) curve analysis, and the optimal value was 436 U/mL, with a sensitivity of 75.0% and a specificity of 61.9% (area under the curve [AUC]: 0.696, 95% CI: 0.594–0.785; *p* < 0.001) (Fig. 3d).

In category 3, contrary to categories 1 and 2, there was no significant difference in the baseline KL-6 level between patients with (*n* = 96) and without TR-ILD (*n* = 187) (310.1 vs. 337.7; *p* = 0.374) (see Supplementary Fig. S2a online). RP was the most common subtype of TR-ILD in category 3 (*n* = 75). However, the median serum KL-6 level was not different among patients with DILD, RP, and AE-ILD (353.7 vs. 281.9 vs. 761.5; *p* = 0.178), although patients with AE-ILD had the highest median serum KL-6 level at baseline (see Supplementary Fig. S2b online). In addition, the median serum KL-6 level at baseline did not correlate with the severity of TR-ILD (284.5 in grade 1 vs. 392.3 in grades 2–4; *p* = 0.967) (see Supplementary Fig. S2c-d online). The cut-off baseline serum KL-6 value for predicting severe TR-ILD in category 3 was 302.4 U/mL with a sensitivity of 76.9% and a specificity of 55.7% (AUC: 0.625, 95% CI 0.521–0.722; *p* = 0.057) (see Supplementary Fig. S2e online).

### Risk factors for the development of severe TR-ILD

**Figure 4:**
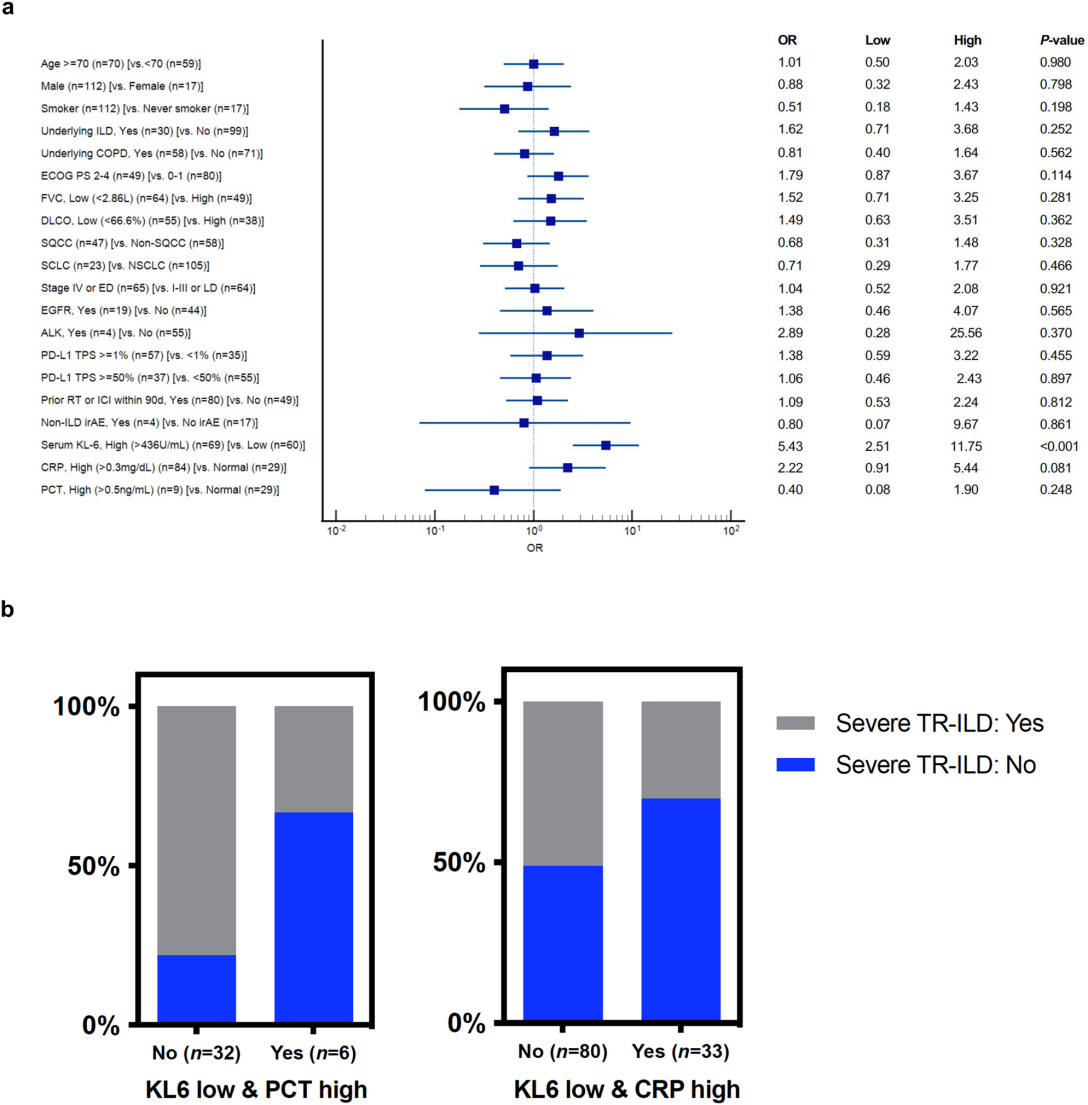
Serum KL-6 as a biomarker for predicting severe TR-ILD in patients with lung cancer (categories 1 and 2). (**a**) Forest plots for risk factors of severe TR-ILD. (**b**) Stacked bar plot for exclusion of severe TR-ILD according to serum KL-6, PCT, and CRP levels at the time of ILD events. *TR-ILD* Treatment-related interstitial lung disease, *OR* Odds ratio, *ILD* Interstitial lung disease, *COPD* Chronic obstructive pulmonary disease, *ECOG* Eastern Cooperative Oncology Group, *PS* Performance status, *FVC* Forced vital capacity, *DLCO* Diffusing capacity of lungs for carbon monoxide, *SQCC* Squamous cell carcinoma, *SCLC* Small cell carcinoma, *NSCLC* Non-small cell lung carcinoma, *ED* Extensive disease, *LD* Limited disease, *EGFR* Epidermal growth factor receptor, *ALK* Anaplastic lymphoma kinase, *PD-L1* Programmed death-ligand 1, *TPS* Tumor proportional score, *RT* Radiation therapy, *ICI* Immune checkpoint inhibitor, *irAE* Immune-related adverse event, *CRP* C-reactive protein, *PCT* Procalcitonin.

In the univariate analysis, a high serum KL-6 level (> 436 U/mL) and a high CRP level (> 0.3 mg/dL) were candidate risk factors for developing severe TR-ILD among patients with lung cancer of categories 1 and 2 (Fig. 4a). In the multivariate analysis, a high serum KL-6 level was an independent risk factor for severe TR-ILD (odds ratio [OR]: 5.51, 95% CI: 2.41–12.60; *p* < 0.001). Furthermore, in categories 1 and 2, a low serum KL-6 level (≤ 436 U/mL) with a high procalcitonin (PCT) level (> 0.5 ng/mL) was associated with non-ILD respiratory disease or non-severe TR-ILD rather than severe TR-ILD (OR: 0.14, 95% CI: 0.21–0.93; *p* = 0.042) (Fig. 4b). Similarly, a low serum KL-6 level (≤ 436 U/mL) with a high CRP level was also associated with non-ILD respiratory disease or non-severe TR-ILD rather than severe TR-ILD (OR: 0.41, 95% CI: 0.18–0.98; *p* = 0.045) (Fig. 4b).

In the univariate analysis of baseline characteristics in category 3, prior RT or ICI therapy within 90 days before starting new cancer treatment (OR: 2.19, 95% CI 0.94–5.10; *p* = 0.069) and a high serum KL-6 level at baseline (> 302.4 U/mL) (OR: 2.62, 95% CI 1.02–6.73; *p* = 0.046) were associated with the development of severe TR-ILD (see Supplementary Fig. S3a online). In multivariate analysis, a high serum KL-6 level at baseline was a significant risk factor for severe TR-ILD (OR: 2.67, 95% CI 1.03–6.90; *p* = 0.043).

### Serum KL-6 levels and prognosis

Survival follow-up was initiated on the date of the first serum KL-6 measurement. On the data cut-off date (February 22, 2022), the median follow-up duration of patients with lung cancer was 18.7 months (95% CI 17.0–20.3). Death, hopeless discharge, or loss of follow-up occurred in 178 patients (42.2%) (Table 1), and the median overall survival (OS) after the first serum KL-6 test was 11.9 months (95% CI 8.9–15.0).

**Figure 5:**
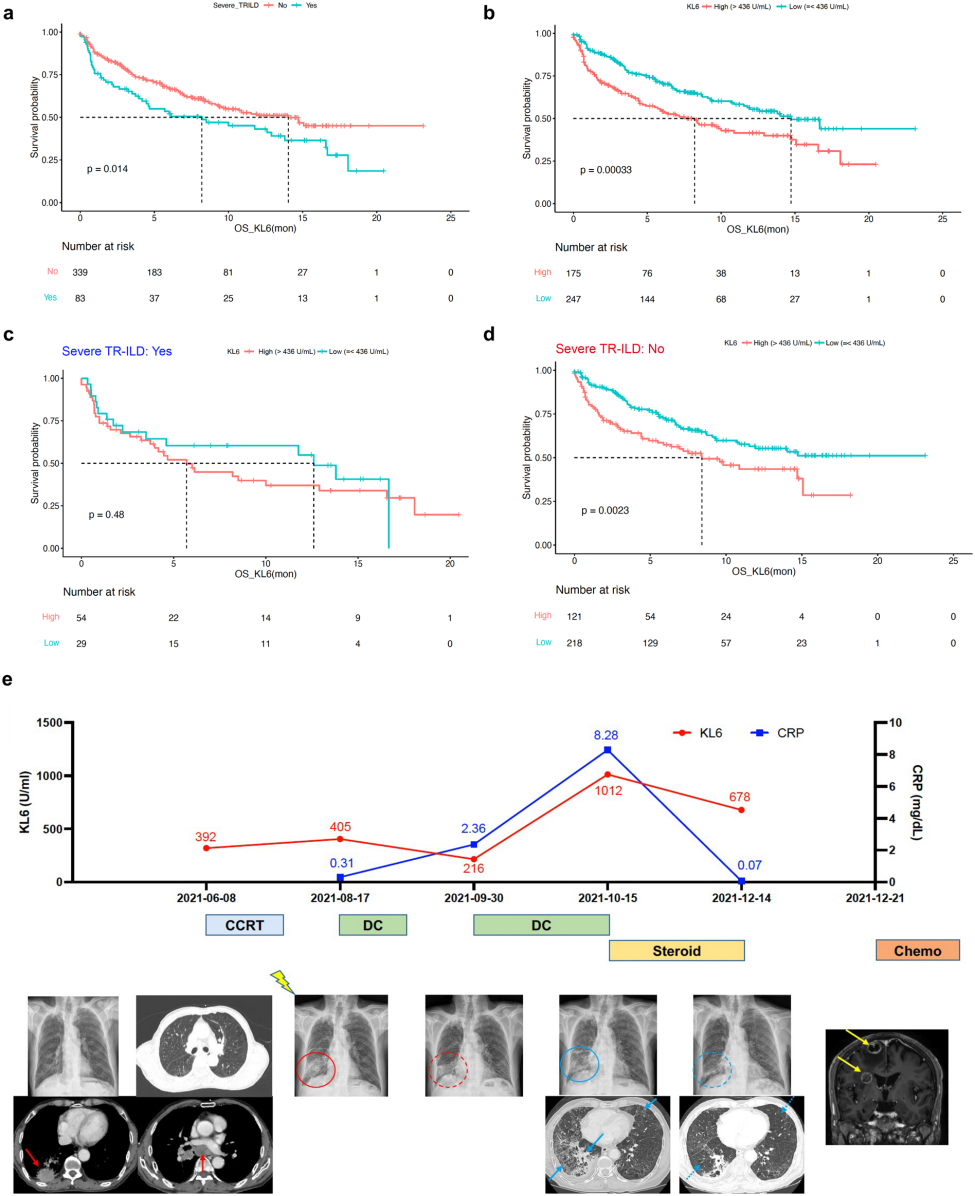
Serum KL-6 as a prognostic biomarker in patients with lung cancer. (**a**) Kaplan–Meier survival curve from the first measurement of serum KL-6 to the events according to the presence of severe TR-ILD in overall patients with lung cancer. (**b**–**d**) Kaplan–Meier survival curve according to high (> 436 U/mL) and low (≤ 436 U/mL) serum KL-6 levels in (**b**) overall patients with lung cancer, (**c**) patients with and (**d**) without severe TR-ILD. (**e**) A representative case of longitudinal monitoring of serum KL-6. This patient with stage-IIIC SQCC of the right lower lobe and mediastinal lymph nodes (red arrows) received durvalumab after CCRT. After 2 weeks, a consolidation in the right lung field developed as shown on his X-ray (red circle), which was consistent with a slight increase in his serum KL-6. During a month-long cessation of durvalumab, the consolidation improved (dashed red circle), and the serum KL-6 level decreased. After durvalumab was restarted, the X-ray appearance of bilateral ground-glass opacities mixed with consolidation was aggravated (blue circle and blue arrows), and the serum KL-6 level markedly increased. The imaging findings resolved after the withdrawal of durvalumab and the establishment of steroid treatment (dashed blue circle and arrows), and the serum KL-6 level gradually declined. However, on restaging workup, multiple brain metastases were detected (yellow arrows), and he underwent subsequent gamma knife radiosurgery and systemic chemotherapy. OS: Overall survival, SQCC: Squamous cell carcinoma, CCRT: Concurrent chemoradiotherapy, DC: Durvalumab consolidation.

Patients with severe TR-ILD showed a significantly worse median OS than those without severe TR-ILD (8.2 months, 95% CI 1.3–15.1 vs. 14.0 months, 95% CI not available; *p* = 0.014) (Fig. 5a). Furthermore, the median OS was significantly shorter among patients with lung cancer with high serum KL-6 levels (> 436 U/mL; 8.2 months, 95% CI 5.3–11.1) than among those with low serum KL-6 levels (≤ 436 U/mL; 14.7 months, 95% CI 11.0–18.4), with a HR of 1.70 (95% CI 1.27–2.28; *p* < 0.001) (Fig. 5b). However, there was no significant difference in survival according to high and low serum KL-6 levels in patients with severe TR-ILD (5.7 months, 95% CI 3.4–8.0 vs. 12.6 months, 95% CI 2.9–22.3; *p* = 0.478) (Fig. 5c), whereas a high serum KL-6 level was associated with worse OS in patients without severe TR-ILD (8.4 months, 95% CI 4.9–11.9 vs. not reached; *p* = 0.002) (Fig. 5d).

In patients whose serum KL-6 levels were tested before starting lung cancer treatment (category 3), events of interest occurred in 108 (38.2%). When the cut-off value of baseline serum KL-6 in category 3 was applied (see Supplementary Fig. S2e online), the median OS was significantly shorter among patients with baseline high serum KL-6 levels (> 302.4 U/mL; 9.8 months, 95% CI 6.1–13.5) than among those with low serum KL-6 levels (≤ 302.4 U/mL; 16.7 months, 95% CI 9.5–23.8), with a HR of 1.53 (95% CI 1.03–2.28; *p* = 0.034) (see Supplementary Fig. S3b online). However, in the same manner as overall patients with lung cancer, there was no significant difference in survival according to baseline high and low serum KL-6 levels in patients with severe TR-ILD (12.9 months, 95% CI 6.9–18.9 vs. 16.7 months, 95% CI not available; *p* = 0.896) (see Supplementary Fig. S3c online), whereas a high serum KL-6 level was associated with worse OS in patients without severe TR-ILD (9.3 months, 95% CI 6.3–12.3 vs. not reached; *p* = 0.030) (see Supplementary Fig. S3d online).

Although serum KL-6 levels were tested once at the time of ILD events or baseline before starting new treatment in most patients, longitudinal monitoring with serum KL-6 levels during treatment was available in several cases (Fig. 5e). In a case of a 75-year-old man with stage IIIC (T3N3M0) squamous cell carcinoma of the right lower lobe and mediastinal lymph nodes (red arrows), durvalumab, an anti-PD-L1 ICI, was administered as a consolidation treatment after the completion of CCRT without progression. On the baseline CT scan before CCRT, the patient had mild bilateral paraseptal emphysema adjacent to the pleura and had no underlying ILD at the time of lung cancer diagnosis. He complained of grade-2 exertional dyspnea on the mMRC (modified Medical Research Council) scale^22^ 2 weeks after initiating durvalumab, and an area of consolidation was revealed in the right lung on his chest X-ray (red circle), concordant with a slight increase in his serum KL-6 level (405 U/mL) compared to baseline level (392 U/mL), suggesting pneumonitis. During a month-long cessation of durvalumab, the consolidation on repeat X-ray examinations improved (dashed red circle), coinciding with an improvement in his symptoms, while his serum KL-6 level decreased to 216 U/mL. After the resumption of durvalumab treatment, his respiratory symptoms (grade-3 dyspnea on the mMRC scale) and X-ray features of bilateral ground-glass opacities mixed with consolidation were aggravated (blue circle and blue arrows), and his serum KL-6 level markedly increased to 1,012 U/mL, reflecting a recurrence of severe TR-ILD. His respiratory symptoms and imaging findings (dashed blue circle and arrows) resolved after durvalumab was withdrawn and steroid treatment was initiated, and his serum KL-6 level gradually declined to 678 U/mL. However, distant metastases (brain; yellow arrows) were detected during the restaging workup, probably due to treatment interruption. After subsequent gamma knife radiosurgery for brain metastases and systemic chemotherapy for 3 months, he was transferred to a hospice facility in July 2022, and follow-up was terminated.

## Discussion

This retrospective cohort study included patients with lung cancer who underwent serum KL-6 testing, which was conducted when an ILD event was suspected or before the initiation of lung cancer treatment. We investigated the usefulness of serum KL-6 levels in the diagnosis and severity assessment of TR-ILD and in predicting TR-ILD development and survival in patients with lung cancer. A high serum KL-6 level was an independent risk factor for severe TR-ILD, and a low serum KL-6 level with a high PCT or CRP level could exclude the diagnosis of severe TR-ILD, rather favoring non-ILD respiratory disease or non-severe TR-ILD. Furthermore, severe TR-ILD was a significant negative prognostic factor, and patients with baseline high serum KL-6 levels had worse OS than those with low serum KL-6 levels, especially in patients without TR-ILD. This study demonstrated that serum KL-6 may represent a biomarker for diagnosing, managing, and predicting TR-ILD in patients with lung cancer.

KL-6 has been used and validated as a potential biomarker for diagnosing and indicating the severity and progression of ILD, particularly in idiopathic interstitial pneumonia, including idiopathic pulmonary fibrosis (IPF)^23–26^. In several meta-analyses, elevated serum KL-6 levels have been associated with disease activity, severity, and survival in the context of ILD^16, 27^; continuously increasing serum KL-6 levels have been demonstrated to indicate poor clinical outcomes^16^. In patients with connective tissue disease, serum KL-6 levels were higher in patients with ILD than in those without ILD, with serum KL-6 levels negatively correlated with lung function (FVC and DLCO) and positively correlated with the extent of ILD^17^. In the present study, the median serum KL-6 level was a useful biomarker for diagnosing TR-ILD, and it was significantly higher in patients who developed ILD than in those without ILD. Most patients without lung cancer developed ILD as an acute exacerbation of underlying idiopathic ILD (see Supplementary Table S1 online), including IPF, nonspecific interstitial pneumonitis (NSIP), cryptogenic organizing pneumonia (COP), and connective tissue disease–associated ILD. Most serum KL-6 levels in patients without lung cancer were measured when respiratory symptoms deteriorated and ILD or non-ILD respiratory disease was suspected. However, this study was not designed to collect data on baseline KL-6 levels at the time of diagnosis of patients with idiopathic ILD, and we did not focus on the validation of serum KL-6 levels as a predictive and prognostic biomarker of idiopathic ILD in patients without lung cancer.

In contrast to idiopathic ILD, the value of serum KL-6 for predicting the development of TR-ILD has not been verified in patients with lung cancer. In a retrospective Japanese study^28^, the proportion of patients with high serum KL-6 levels (> 500 U/mL) was higher among patients with lung cancer with ILD (73.5%) than among those without ILD (33.7%). In a systematic review and meta-analysis, higher levels of KL-6 at baseline were associated with AE-ILD following lung cancer resection^29^. However, in the reports of these studies, the exact time point (baseline or onset) and reason for measuring serum KL-6 levels were not described^28^, or the causes or subtypes of ILD were not defined^28, 29^. In the present study, we divided patients into three subgroups according to the timing and purpose of serum KL-6 testing to verify the significance of serum KL-6 in the diagnosis and severity assessment of ILD at its onset (categories 1 and 2) and in predicting the occurrence of ILD before lung cancer treatment at baseline (category 3). Furthermore, ILD development was defined as a treatment-related event (TR-ILD), and the subtypes of ILD were categorized as DILD, RP, and AE-ILD, according to the cause of TR-ILD. This study showed the same trend as previous studies of ILD in patients without lung cancer. A high serum KL-6 level (> 436 U/mL) was an independent predictor of the development of severe TR-ILD at the time of its onset, and a low serum KL-6 level with a high serum PCT or CRP level could exclude severe TR-ILD. In addition, the median serum KL-6 level was significantly higher in the DILD or AE-ILD subgroups than in the RP or non-ILD subgroups. These results demonstrated that serum KL-6 levels could be useful as a predictive biomarker for differentiating between severe TR-ILD and non-severe TR-ILD or non-ILD respiratory disease and for assessing the severity of TR-ILD in patients with lung cancer under treatment. However, the assessment of the tests with KL-6 and inflammatory markers (PCT and CRP) was based on some cases, and a large-scale prospective study might be needed to verify the usefulness of this combination of tests.

Serum KL-6 levels have been reported to be associated with the development and clinical course of DILD and RP. In several previous studies, elevated KL-6 levels have been observed in most patients with DILD^30^, and serum KL-6 levels have been shown to change over time in response to treatment^18, 30^. In patients with RP, post-RT serum KL-6 levels were increased relative to pretreatment levels during or after thoracic RT^31, 32^. However, regarding discrimination between subtypes of TR-ILD, caution should be exercised with respect to the positive predictive value of the KL-6 assay, as elevated serum KL-6 levels can vary according to radiologic patterns and by the extent and severity of ILD. In the aforementioned studies, serum KL-6 levels were increased in patients with DILD with diffuse alveolar damage, chronic interstitial pneumonia, or acute interstitial pneumonia patterns, but not in those with bronchiolitis obliterans organizing pneumonia, eosinophilic pneumonia, or hypersensitivity pneumonitis patterns^18, 30^. In addition, serum KL-6 levels increased from baseline in patients with severe RP, whereas there was no significant change in patients with localized RP^31^. In the present study, the median serum KL-6 levels were significantly higher in the DILD or AE-ILD subgroups than in the RP or non-ILD subgroups, whereas the patients with RP were not significantly different from those with non-ILD lung cancer in this regard. RP can be categorized as a localized subtype of TR-ILD compared to DILD or AE-ILD. Thus, taken together with the imaging findings, temporal causality, and serum KL-6 level in the case presentation (Fig. 5e), the first event of pneumonitis associated with a slight increase in serum KL-6 should be assumed to be RP. Increased serum KL-6 levels are correlated with increased regeneration of alveolar type II pneumocytes and enhanced permeability following the destruction of the air-blood barrier^14, 15, 33^. Therefore, the results of this study are consistent with those of previous studies, suggesting that they can be steadily translated into real-world clinical practice.

Few studies have investigated KL-6 as a prognostic tumor marker in patients with lung cancer. In a surgical cohort study, immunohistochemical findings in NSCLC were correlated with serum KL-6 levels, and a high serum KL-6 level was associated with a poor prognosis in patients with NSCLC who had undergone curative surgery^34^. In a retrospective Japanese study, elevated serum KL-6 levels were not associated with prognosis in patients with lung cancer with ILD; however, it was one of the unfavorable prognostic factors in individuals without ILD^28^. Similarly, in the present study, high serum KL-6 levels were not a significant prognostic factor in patients with severe TR-ILD. We assume that the prognosis of patients with severe TR-ILD may be associated with complex factors and the origins of the elevation in serum KL-6 level. Serum KL-6 levels were associated with the baseline characteristics of lung cancer, including comorbidity (underlying ILD), ECOG PS score, histology, stage (advanced), baseline lung function (FVC, DLCO), and a tumor marker (CEA). Factors related to comorbidities, tumor burden, or treatment modality may affect the clinical course of patients with TR-ILD after the events. The value of baseline serum KL-6 for predicting the development of TR-ILD was diluted in category 3, probably because of variations in comorbidities, stage, and histology in this group of patients. However, in patients without severe TR-ILD, high serum KL-6 levels before cancer treatment were associated with shorter OS. We assume that serum KL-6 levels reflect the cancer-related parameters, such as comorbidity or tumor burden, and could aid in predicting the prognosis of patients without TR-ILD. Therefore, the current study might suggest the potential of serum KL-6 as a baseline prognostic biomarker for patients with lung cancer with treatment plans. However, further studies are needed to validate the clinical implications of predicting prognosis in patients with TR-ILD.

This study has several limitations that warrant discussion. First, as the clinical data of patients with lung cancer were retrospectively collected, given the general distribution in the lung cancer population, there were imbalances in the subgroups concerning pulmonary function, type of initial therapy, the purposes of KL-6 testing, and ILD subtypes (Table 1). Second, there were insufficient instances of repeated tests and serial follow-up of serum KL-6 levels. Although we provided potential evidence of successful monitoring of serum KL-6 levels in a representative case (Fig. 5e), it was impossible to objectively demonstrate the utility of serum KL-6 monitoring with the small sample size, as the progress of ILD changed. In an ILD cohort study^21^, serial blood samples were collected from patients with IPF with or without lung cancer before initiating antifibrotic therapy (nintedanib) and after 12 to 24 months. In this cohort study, baseline serum KL-6 levels were higher in patients with IPF with lung cancer than in those without lung cancer, and an elevation in serum KL-6 levels was correlated with a significant decline in FVC during follow-up. A large prospective cohort study that includes specific lung cancer populations stratified by stage, histology, treatment modality, or the timing of repeat KL-6 testing is needed to verify the usefulness of serial KL-6 monitoring during or after cancer treatment.

In conclusion, the present study demonstrated that a high serum KL-6 level at the onset of an ILD event was an independent predictor of the development of severe TR-ILD, and a combination of serum KL-6 and PCT or CRP levels was useful for distinguishing between severe TR-ILD and non-severe TR-ILD or non-ILD respiratory disease. Serum KL-6 levels were associated with several baseline characteristics of lung cancer and causality-related subtypes of TR-ILD. Furthermore, severe TR-ILD was associated with worse survival in patients with lung cancer, and a high serum KL-6 level before treatment was a significant negative prognostic factor, especially in patients without TR-ILD. These findings may provide valuable insights into the clinical vigilance and management of TR-ILD with timely serum KL-6 testing during lung cancer treatment. Future larger-scale prospective studies are warranted to validate the clinical significance and prognostic potential of serum KL-6 levels in patients with lung cancer who undergo treatment that has the potential to cause ILD.

## Methods

### Patients and the assessment of ILD

We hypothesized that the high KL-6 level measured at the onset of ILD events or at baseline before starting treatment is associated with the development and severity of TR-ILD in patients with lung cancer and can aid in differentiating TR-ILD from other diseases similar to ILD, which occur during or after treatment. We recruited patients with or without lung cancer who were tested for serum KL-6 at Chonnam National University Hwasun Hospital between January 2020 and January 2022. Computed tomographic images were evaluated for the presence of ILD at the time of the event or before lung cancer treatment. Two radiology and pulmonology experts checked the images to confirm a diagnosis of ILD. TR-ILD was clinically diagnosed on the basis of the worsening of dyspnea, newly developed pulmonary interstitial infiltrates on chest CT scan after the initiation of lung cancer treatment, and exclusion of alternative diseases that could cause respiratory distress, such as infection, tumor progression, pneumothorax, heart failure or pulmonary embolism. When the development of TR-ILD was suspected, chest CT scan, electrocardiography, serum cardiac enzyme test, and microbiologic evaluation, including sputum culture, blood culture, urine antigen test, or polymerase chain reaction test were performed. Tumor progression was excluded on the basis of chest CT findings, physical examinations, and tumor markers such as CEA or pro-gastrin-releasing peptide (ProGRP). ILD was categorized as follows: DILD, RP, or AE-ILD. Subtypes of ILD were defined and differentiated based on the temporal correlation with treatment modalities, the area of radiation exposure, the extent of the ILD on a chest CT scan, and the presence of preexisting ILD. AE-ILD was defined in patients who had previous medical records or relevant CT images of ILD, and when a chest CT scan demonstrated the radiologic evidence of disease progression in pre-existing ILD, following the criteria proposed by the American Thoracic Society/European Respiratory Society/Japanese Respiratory Society/Latin American Thoracic Association Clinical Practice Guideline published in 2022^35^. When the diagnosis of ILD subtypes was not specified, the final diagnosis was made by the consensus of two radiology and pulmonology experts.

### Data collection

We conducted a retrospective cohort study using a medical records database and the Chonnam National University Hwasun Hospital lung cancer registry, with data collected by pulmonology specialists. The database contains information on personal details (e.g., patient identifiers, date of birth, and sex), medical history (e.g., family and personal history of cancer, smoking status, comorbidities), lung cancer characteristics (e.g., stage at registry enrollment, ECOG PS), pulmonary comorbidities, pulmonary function, EGFR mutation, anaplastic lymphoma kinase (ALK) rearrangement, and PD-L1 tumor proportional score. The ECOG PS score was categorized as good (0–1) or poor (2–4). Additionally, longitudinal information on the treatment of lung cancer and ILD was available in the database and registry through institutional electronic health records, which allowed this study to capture the type, dose, and date of treatment administration of treatment at the patient level. At the time of analysis after data collection, all patient information was anonymized. Data on pulmonary function, inflammatory markers (such as CRP and PCT), and tumor markers (such as CEA) were based on the results of tests performed around the time point of serum KL-6 measurements. Thus, the timing of those tests in categories 1 and 2 was at diagnosis or around the onset of the events, and in category 3 was at diagnosis or before cancer treatment. The variables reflecting pulmonary function were forced expiratory volume within 1 s (FEV1), FVC, and DLCO. In patients confirmed to have developed ILD, we assessed the severity of TR-ILD according to the Common Terminology Criteria for Adverse Events (version 5.0), and we defined severe TR-ILD as grade 2 or higher.

The study was conducted in accordance with the Declaration of Helsinki (as revised in 2013) and Good Clinical Practice guidelines. This study was approved by the Institutional Review Board of Chonnam National University Hwasun Hospital (CNUHH-2022-206). The requirement for patient consent was waived by the Institutional Review Board of the Chonnam National University Hwasun Hospital because of the retrospective nature of the study and because the analysis used anonymized clinical data.

### Blood sample collection and measurement of serum KL-6 levels

Serum KL-6 levels (U/mL) were measured using an AU 5800 chemistry analyzer (Beckman Coulter, Brea, CA, USA) with the Nanopia KL-6 assay (Sekisui Medical Co., Ltd., Tokyo, Japan). Serum KL-6 tests may have been repeated, and the results were monitored by attending physicians according to the clinical course of TR-ILD.

### Statistical analysis

To compare serum KL-6 levels between subgroups, we used the *t*-test and one-way ANOVA (analysis of variance) followed by Tukey’s post hoc test. Pearson’s correlation coefficient was used to analyze the associations between serum KL-6 levels and several covariates, such as pulmonary function, tumor markers, and inflammatory markers. ROC curve analysis was performed to determine the optimal serum KL-6 cut-off value for predicting severe TR-ILD. Chi-square and logistic regression analyses were performed to determine risk factors for severe TR-ILD. Survival times were measured from the respective dates when serum KL-6 levels were measured for the first time, and these were estimated for serum KL–6–based subgroups using the Kaplan–Meier method. Univariate and multivariate analyses of survival were performed using Cox proportional hazards modeling. Statistical analysis was performed using SPSS Statistics for Windows, version 27 (IBM Corp., Armonk, NY, USA) and R version 4.2.3 (R Foundation for Statistical Computing, Vienna, Austria)^36^. A *p*-value < 0.05 was considered statistically significant.

### Supplementary Information

Supplementary Information.

## Data Availability

The data presented in this study are available on request from the corresponding author. The data are not publicly available due to institutional data-sharing restrictions.

## Abbreviations

KL-6: Krebs von den Lungen-6
TR-ILD: Treatment-related interstitial lung disease
ILD: Interstitial lung disease
ICI: Immune checkpoint inhibitor
EGFR: Epidermal growth factor receptor
NSCLC: Non-small cell lung cancer
PD-1: Programmed death-1
PD-L1: Programmed death ligand-1
CRT: Chemoradiation therapy
CCRT: Concurrent chemoradiation therapy
HR: Hazard ratio
CI: Confidence interval
RP: Radiation-induced pneumonitis
ECOG: Eastern cooperative oncology group
PS: Performance status
FVC: Forced vital capacity
DLCO: Diffusing capacity of lungs for carbon monoxide
CEA: Carcinoembryonic antigen
RT: Radiation therapy
DILD: Drug-induced ILD
AE-ILD: Acute exacerbation of underlying ILD
PCT: Procalcitonin
mMRC: Modified medical research council
IPF: Idiopathic pulmonary fibrosis
NSIP: Nonspecific interstitial pneumonitis
COP: Cryptogenic organizing pneumonia
ALK: Anaplastic lymphoma kinase
FEV1: Forced expiratory volume within 1 s
ANOVA: Analysis of variance

## References

[1] Sung H (2021). Global cancer statistics 2020: GLOBOCAN estimates of incidence and mortality worldwide for 36 cancers in 185 countries. CA Cancer J. Clin..

[2] Hong S (2021). Cancer statistics in Korea: Incidence, mortality, survival, and prevalence in 2018. Cancer Res. Treat.

[3] Chang CH, Chang YC (2022). Comparing the therapeutic efficacies of lung cancer: Network meta-analysis approaches. Int. J. Environ. Res. Public Health.

[4] Wu Y-L (2020). Osimertinib in resected EGFR-mutated non–small-cell lung cancer. N. Engl. J. Med..

[5] Forde PM (2022). Neoadjuvant nivolumab plus chemotherapy in resectable lung cancer. N. Engl. J. Med..

[6] Felip E (2021). Adjuvant atezolizumab after adjuvant chemotherapy in resected stage IB-IIIA non-small-cell lung cancer (IMpower010): A randomised, multicentre, open-label, phase 3 trial. Lancet.

[7] O'Brien M (2022). Pembrolizumab versus placebo as adjuvant therapy for completely resected stage IB-IIIA non-small-cell lung cancer (PEARLS/KEYNOTE-091): An interim analysis of a randomised, triple-blind, phase 3 trial. Lancet Oncol..

[8] Spigel DR (2022). Five-year survival outcomes from the PACIFIC trial: Durvalumab after chemoradiotherapy in stage III non-small-cell lung cancer. J. Clin. Oncol..

[9] Saito G (2021). Real-world survey of pneumonitis and its impact on durvalumab consolidation therapy in patients with non-small cell lung cancer who received chemoradiotherapy after durvalumab approval (HOPE-005/CRIMSON). Lung Cancer.

[10] Liu X, Shao C, Fu J (2021). Promising biomarkers of radiation-induced lung injury: A review. Biomedicines.

[11] Li Y (2022). Eosinophil as a biomarker for diagnosis, prediction, and prognosis evaluation of severe checkpoint inhibitor pneumonitis. Front. Oncol..

[12] Lin X (2021). Peripheral blood biomarkers for early diagnosis, severity, and prognosis of checkpoint inhibitor-related pneumonitis in patients with lung cancer. Front. Oncol..

[13] Schoenfeld JD (2019). Pneumonitis resulting from radiation and immune checkpoint blockade illustrates characteristic clinical, radiologic and circulating biomarker features. J. Immunother. Cancer.

[14] Kohno N (1993). KL-6, a mucin-like glycoprotein, in bronchoalveolar lavage fluid from patients with interstitial lung disease. Am. Rev. Respir. Dis..

[15] Ishikawa N, Hattori N, Yokoyama A, Kohno N (2012). Utility of KL-6/MUC1 in the clinical management of interstitial lung diseases. Respir. Investig..

[16] Zhang T, Shen P, Duan C, Gao L (2021). KL-6 as an immunological biomarker predicts the severity, progression, acute exacerbation, and poor outcomes of interstitial lung disease: A systematic review and meta-analysis. Front. Immunol..

[17] Lee JS (2019). Serum KL-6 levels reflect the severity of interstitial lung disease associated with connective tissue disease. Arthritis Res. Ther..

[18] Kawase S (2011). Change in serum KL-6 level from baseline is useful for predicting life-threatening EGFR-TKIs induced interstitial lung disease. Respir. Res..

[19] Nakahama K (2023). Clinical significance of KL-6 in immune-checkpoint inhibitor treatment for non-small cell lung cancer. Cancer Chemother. Pharmacol..

[20] Iwata H (2011). Correlation between the serum KL-6 level and the grade of radiation pneumonitis after stereotactic body radiotherapy for stage I lung cancer or small lung metastasis. Radiother. Oncol..

[21] d'Alessandro M (2021). Serum concentrations of KL-6 in patients with IPF and lung cancer and serial measurements of KL-6 in IPF patients treated with antifibrotic therapy. Cancers.

[22] Mahler DA, Wells CK (1988). Evaluation of clinical methods for rating dyspnea. Chest.

[23] Zheng P (2018). Diagnostic value of KL-6 in idiopathic interstitial pneumonia. J. Thorac. Dis..

[24] Yokoyama A (2006). Prognostic value of circulating KL-6 in idiopathic pulmonary fibrosis. Respirology.

[25] Bergantini L (2020). Utility of serological biomarker' panels for diagnostic accuracy of interstitial lung diseases. Immunol. Res..

[26] Wakamatsu K (2017). Prognostic value of serial serum KL-6 measurements in patients with idiopathic pulmonary fibrosis. Respir. Investig..

[27] Zhang H (2020). Diagnostic and prognostic predictive values of circulating KL-6 for interstitial lung disease: A PRISMA-compliant systematic review and meta-analysis. Medicine.

[28] Miyazaki K (2010). Serum KL-6 levels in lung cancer patients with or without interstitial lung disease. J. Clin. Lab. Anal..

[29] Hao X (2022). Risk factors for acute exacerbation of interstitial lung disease following lung cancer resection: A systematic review and meta-analysis. Interact. Cardiovasc. Thorac. Surg..

[30] Ohnishi H (2003). Circulating KL-6 levels in patients with drug induced pneumonitis. Thorax.

[31] Goto K (2001). Serum levels of KL-6 are useful biomarkers for severe radiation pneumonitis. Lung Cancer.

[32] Hara R, Itami J, Komiyama T, Katoh D, Kondo T (2004). Serum levels of KL-6 for predicting the occurrence of radiation pneumonitis after stereotactic radiotherapy for lung tumors. Chest.

[33] Ohnishi H (2002). Comparative study of KL-6, surfactant protein-A, surfactant protein-D, and monocyte chemoattractant protein-1 as serum markers for interstitial lung diseases. Am. J. Respir. Crit. Care Med..

[34] Tanaka S (2012). Krebs von den Lungen-6 (KL-6) is a prognostic biomarker in patients with surgically resected nonsmall cell lung cancer. Int. J. Cancer.

[35] Raghu G (2022). Idiopathic pulmonary fibrosis (an update) and progressive pulmonary fibrosis in adults: An official ATS/ERS/JRS/ALAT clinical practice guideline. Am. J. Respir. Crit. Care Med..

[36] R Core Team. A Language and Environment for Statistical Computing. *R Foundation for Statistical Computing, Vienna, Austria* (2021).

