# Supplementary material for: Serum KL-6 levels predict the occurrence and severity of treatment-related interstitial lung disease in lung cancer
Source: Sci Rep. 2023 Oct 23;13:18126. doi: 10.1038/s41598-023-45170-8 (PMC10593856; doi:10.1038/s41598-023-45170-8)
Supplement: Supplementary file 1 — Supplementary Information. [file 41598_2023_45170_MOESM1_ESM.pdf]

Serum KL-6 levels predict the occurrence and severity of treatment-related interstitial lung disease in lung cancer

Hwa Kyung Park[1,2], Chang-Seok Yoon[1,2], Young-Ok Na[2,3], Jae-Kyeong Lee[2,3], Hyung-Joo Oh[1,2], Ha-Young Park[2,4], Bo-Gun Kho[2,3], Tae-Ok Kim[2,3], Hong-Joon Shin[2,3], Yong-Soo Kwon[2,3], In-Jae Oh[1,2], Yu-Il Kim[2,3], Sung-Chul Lim[2,3], Young-Chul Kim[1,2] and Cheol-Kyu Park[1,2,*]

Supplementary Information Lists

Table S1. Comparison of baseline characteristics between patients with and without lung cancer.

Supplementary Fig. S1. Comparison of serum KL-6 levels in the overall population.

Supplementary Fig. S2. Baseline serum KL-6 and TR-ILD in patients with lung cancer (category 3).

Supplementary Fig. S3. Baseline serum KL-6 as a biomarker for predicting severe TR-ILD and prognosis in patients with lung cancer (category 3).

Supplementary Information

Table S1. Comparison of baseline characteristics between patients with and without lung cancer.

Characteristic	Total (*n*=1,297)	Non-LC (*n*=875)	LC (*n*=422)	*P*-value
**Age**	71 (18-96)	73 (18-96)	70 (36-86)	<0.001
**Sex**				<0.001
Female	321 (24.7)	273 (31.2)	48 (11.4)	
Male	976 (75.3)	602 (68.8)	374 (88.6)	
**Smoking**				<0.001
Never smoker	544 (41.9)	482 (55.1%)	62 (14.7)	
Current smoker	279 (21.5)	126 (14.4%)	153 (36.3)	
Ex-smoker	474 (36.5)	267 (30.5%)	207 (49.0)	
**Comorbidity**				
ILD	718 (55.4)	674 (77.0)	44 (10.4)	<0.001
COPD	433 (33.4)	219 (25.0)	214 (50.7)	<0.001
**ECOG PS score**				<0.001
0	220 (17.0)	205 (23.4)	15 (3.5)	
1	690 (53.2)	392 (44.8)	298 (70.6)	
2	175 (13.5)	78 (8.9)	97 (23.0)	
3	93 (7.1)	85 (9.7)	8 (1.9)	
4	119 (9.2)	115 (13.1)	4 (1.0)	
**Pulmonary function**				
FEV1, L (*n*=779)	2.11 (0.43-3.84)	2.19 (0.43-3.84)	2.03 (0.52-3.80)	0.004
FEV1, % (*n*=779)	75.3 (11.0-148.0)	80.0 (11.0-148.0)	71.1 (20.1-124.5)	<0.001
FVC, L (*n*=779)	2.85 (0.44-6.24)	2.83 (0.44-5.08)	2.86 (1.11-6.24)	0.181
FVC, % (*n*=779)	72.0 (21.0-120.0)	73.0 (21.0-120.0)	71.4 (30.8-116.8)	0.061
DLCO, % (*n*=692)	65.0 (11.0-135.0)	62.0 (11.0-135.0)	66.6 (24.9-133.0)	0.006
**Serum CEA, ng/mL (*n*=373)**	-	-	5.50 (0.88-11450.37)	-
**Serum CRP, mg/dL (*n*=1,134)**	0.80 (0.01-52.57)	0.58 (0.01-38.36)	1.42 (0.02-52.57)	<0.001
**Serum PCT, ng/mL (*n*=300)**	0.20 (0.02-81.8)	0.20 (0.02-81.8)	0.20 (0.03-2.87)	0.940
**Initial diagnosis**				-
Lung cancer	-	-	422 (100.0)	
ILD	-	670 (76.6)	-	
Pneumonia	-	154 (17.6)	-	
Others	-	51 (5.8)	-	
**Histology**				-
ADC	-	-	154 (36.5)	
SQCC	-	-	174 (41.2)	

NSCLC, NOS	-	-	17 (4.0)	
SCLC	-	-	77 (18.2)	
**Stage (TNM 8th)**				-
Early (I-III, LD)	-	-	178 (42.2)	
Advanced (IV, ED)	-	-	244 (57.8)	
**Driver mutation**				-
EGFR (*n*=191)	-	-	34 (17.8)	
ALK (*n*=186)	-	-	14 (7.5)	
ROS1 (*n*=166)	-	-	2 (1.2)	
**PD-L1 IHC (SP263) (*n*=312)**				-
TPS <1%	-	-	120 (38.6)	
TPS ≥1%, <50%	-	-	90 (28.8)	
TPS ≥50%	-	-	102 (32.6)	
**PD-L1 IHC (22C3) (*n*=157)**				-
TPS <1%	-	-	39 (24.8)	
TPS ≥1%, <50%	-	-	56 (35.7)	
TPS ≥50%	-	-	62 (39.5)	
**Initial therapy of LC**				-
Operation	-	-	59 (14.0)	
CCRT	-	-	162 (38.4)	
Systemic chemotherapy	-	-	180 (42.6)	
SBRT or RT alone	-	-	12 (2.8)	
Palliative treatment	-	-	3 (0.7)	
Supportive care	-	-	6 (1.4)	
**IrAE (*n*=270)**			53 (19.6)	
**KL-6 test category**				<0.001
1	355 (27.4)	295 (33.7)	60 (14.2)	
2	647 (49.9)	578 (66.1)	69 (16.3)	
3	283 (21.8)	0 (0.0)	283 (67.1)	
Unknown	12 (0.9)	2 (0.2)	10 (2.4)	
**ILD type**	865 (66.7)	670 (76.6)	195 (46.2)	<0.001
DILD	60 (6.9)	9 (1.3)	51 (26.2)	
RP	120 (13.9)	1 (0.2)	119 (61.0)	
AE-ILD	685 (79.2)	660 (98.5)	25 (12.8)	
**Survival**				0.001
Live	730 (56.3)	486 (55.5)	244 (57.8)	
Death	172 (13.3)	99 (11.3)	73 (17.3)	

Lost F/U or Hopeless discharge	395 (30.4)	290 (33.2)	105 (24.9)	

Values are presented as medians (ranges) or numbers (%). LC: Lung cancer, ILD: Interstitial lung disease, COPD: Chronic obstructive pulmonary disease, ECOG: Eastern Cooperative Oncology Group, PS: Performance status, FEV1: Forced expiratory volume within 1 second, FVC: Forced vital capacity, DLCO: Diffusing capacity of lungs for carbon monoxide, CEA: Carcinoembryonic antigen, CRP: C-reactive protein, PCT: Procalcitonin, ADC: adenocarcinoma, SQCC: squamous cell carcinoma, NSCLC: Non-small cell lung carcinoma, NOS: Not otherwise specified, SCLC: Small cell lung carcinoma; LD: Limited disease (for small cell lung cancer), ED: Extensive disease (for small cell lung cancer), EGFR: Epidermal growth factor receptor, ALK: Anaplastic lymphoma kinase, ROS1: ROS proto-oncogene 1, PD-L1: Programmed death-ligand 1, IHC: Immunohistochemistry, TPS: Tumor proportion score, CCRT: Concurrent chemoradiation therapy, SBRT: Stereotactic body radiation therapy, RT: Radiation therapy, IrAE: Immune-related adverse even, DILD: Drug-induced ILD, RP: Radiation-induced pneumonitis, AE-ILD: Acute exacerbation of underlying ILD, F/U: follow-up.

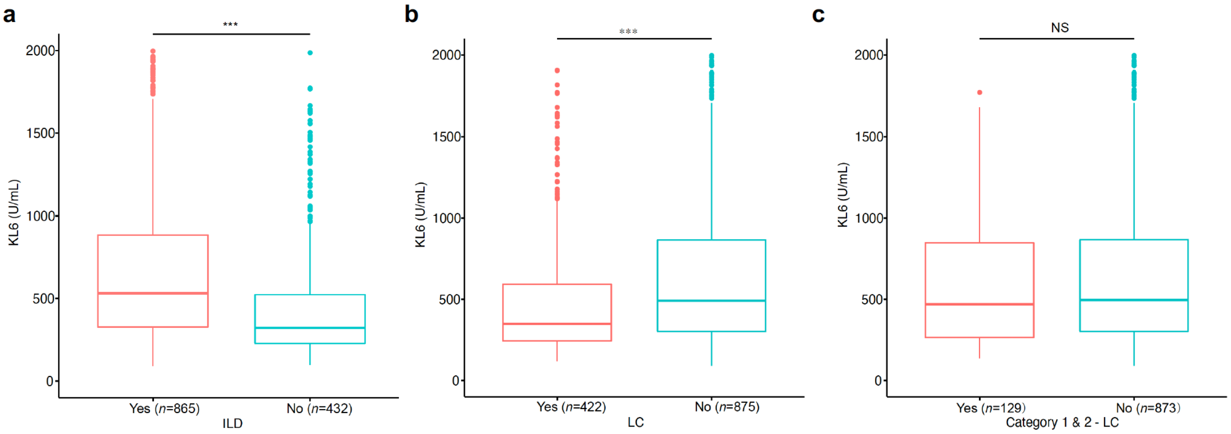

Supplementary Fig. S1. Comparison of serum KL-6 levels in the overall population. (a–c) The box-and-whisker plots show (a) the difference between patients with ILD and without ILD and (b) the difference between patients with lung cancer and without lung cancer overall. (c) The plot shows the difference between patients with and without lung cancer in categories 1 and 2. $^{***}p < 0.001$. NS: Not significant, ILD: Interstitial lung disease, LC: Lung cancer.

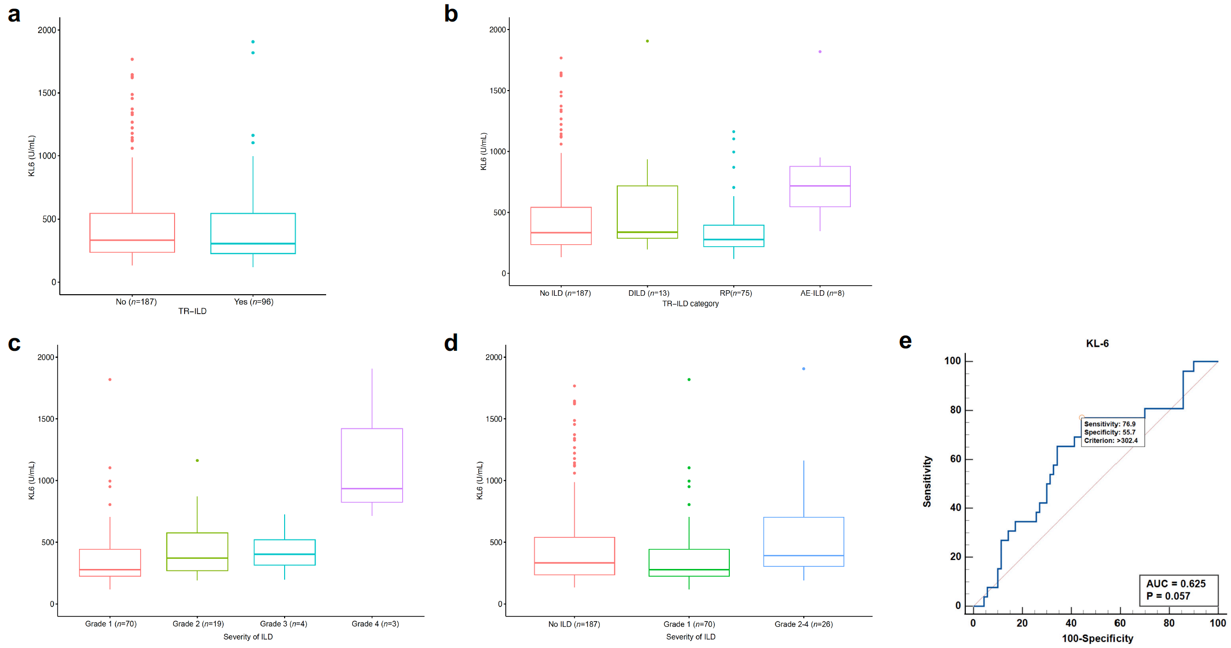

Supplementary Fig. S2. Baseline serum KL-6 and TR-ILD in patients with lung cancer (category 3). (a–d) The box-and-whisker plots show differences in median KL-6 levels according to (a) the presence of TR-ILD, (b) the subtype, and (c–d) the severity of TR-ILD. (e) ROC curve analysis of cut-off baseline serum KL-6 value for predicting severe TR-ILD. TR-ILD: Treatment-related interstitial lung disease, DILD: Drug-induced ILD, RP: Radiation-induced pneumonitis, AE-ILD: Acute exacerbation of underlying ILD, ILD: Interstitial lung disease, ROC: Receiver operating characteristic, AUC: Area under the curve.

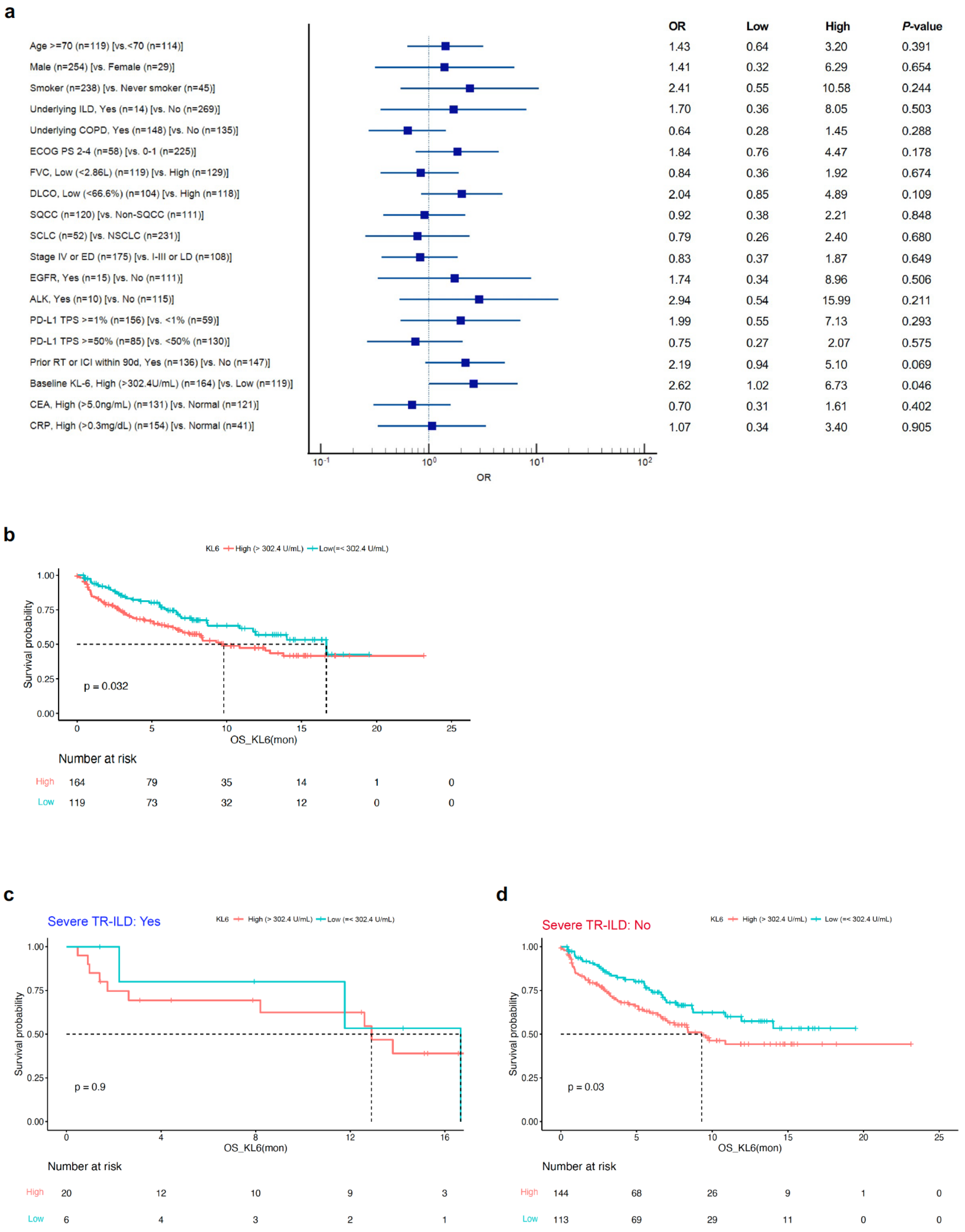

Supplementary Fig. S3. Baseline serum KL-6 as a biomarker for predicting severe TR-ILD and prognosis in patients with lung cancer (category 3). (a) Forest plots for risk factors of severe TR-ILD.

(b–d) Kaplan–Meier survival curve from the first measurement of serum KL-6 to the events according to high (> 302.4 U/mL) and low (≤ 302.4 U/mL) baseline serum KL-6 levels in (b) overall patients with lung cancer of category 3, (c) patients with and (d) without severe TR-ILD. TR-ILD: Treatment-related interstitial lung disease, OR: Odds ratio, ILD: Interstitial lung disease, COPD: Chronic obstructive pulmonary disease, ECOG: Eastern Cooperative Oncology Group, PS: Performance status, FVC: Forced vital capacity, DLCO: Diffusing capacity of the lungs for carbon monoxide, SQCC: Squamous cell carcinoma, SCLC: Small cell carcinoma, NSCLC: Non-small cell lung carcinoma, ED: Extensive disease, LD: Limited disease, EGFR: Epidermal growth factor receptor, ALK: Anaplastic lymphoma kinase, PD-L1: Programmed death-ligand 1, TPS: Tumor proportional score, RT: Radiation therapy, ICI: Immune checkpoint inhibitor, CEA: Carcinoembryonic antigen, CRP: C-reactive protein, OS: Overall survival.
